# A Quantum Dot-Based FLIM Glucose Nanosensor

**DOI:** 10.3390/s19224992

**Published:** 2019-11-16

**Authors:** Consuelo Ripoll, Angel Orte, Lorena Paniza, Maria Jose Ruedas-Rama

**Affiliations:** Department Physical Chemistry, Faculty of Pharmacy, Unidad de Excelencia de Química Aplicada a la Biomedicinay Medioambiente (UEQABM), University of Granada, Campus Cartuja, 18071 Granada, Spain

**Keywords:** quantum dots, fluorescence lifetime imaging, intracellular sensing, nanoparticles, photoluminescence, glucose

## Abstract

In the last few years, quantum dot (QD) nanoparticles have been employed for bioimaging and sensing due to their excellent optical features. Most studies have used photoluminescence (PL) intensity-based techniques, which have some drawbacks, especially when working with nanoparticles in intracellular media, such as fluctuations in the excitation power, fluorophore concentration dependence, or interference from cell autofluorescence. Some of those limitations can be overcome with the use of time-resolved spectroscopy and fluorescence lifetime imaging microscopy (FLIM) techniques. In this work, CdSe/ZnS QDs with long decay times were modified with aminophenylboronic acid (APBA) to achieve QD-APBA conjugates, which can act as glucose nanosensors. The attachment of the boronic acid moiety on the surface of the nanoparticle quenched the PL average lifetime of the QDs. When glucose bonded to the boronic acid, the PL was recovered and its lifetime was enhanced. The nanosensors were satisfactorily applied to the detection of glucose into MDA-MB-231 cells with FLIM. The long PL lifetimes of the QD nanoparticles made them easily discernible from cell autofluorescence, thereby improving selectivity in their sensing applications. Since the intracellular levels of glucose are related to the metabolic status of cancer cells, the proposed nanosensors could potentially be used in cancer diagnosis.

## 1. Introduction

Glucose is the major energy source for the maintenance of cell homeostasis and cell proliferation. Cancer cells primarily rely on glucose and glutamine to supply intermediary metabolism, so the glucose metabolism level reflects the cell’s proliferative status [1]. Moreover, anomalous glucose levels in the blood or physiological fluids are directly related to some diseases, such as diabetes [2], so the development of new and improved glucose sensors is of high interest for biomedical diagnosis and for applications in healthcare products [3]. 

To date, many different glucose sensors have been developed to monitor the changes in glucose concentrations. Enzymatic-based glucose probes have prevailed for decades, being glucose oxidase (GOx) the most commonly used enzyme. The sensing mechanism is based on the oxidation of glucose by molecular oxygen catalyzed by GOx, which generates H_2_O_2_. The first systems were very basic electrochemical sensors [4,5,6] that essentially used amperometric detection, although, more recently, the combination of GOx with new nanomaterials, such as semiconductor nanoparticles [7,8], carbon nanotubes [9], or graphene [10], has produced more sophisticated and highly sensitive glucose sensors.

On the other hand, glucose sensing can also be accomplished with non-enzymatic approaches. Among other sensing systems, boronic acid-based sensors have attracted significant attention in the past two decades. Generally, most boronic acid sensors are developed on the basis of covalent bond forming interactions between boronic acid and diols, which enables the design of a wide range of boronic acid-based saccharide sensors [11]. In particular, synthetic fluorescent sensors with boronic acid groups in their structures are an excellent option for glucose sensing [12]. The use of boronic acid moiety as the quencher of a fluorescent dye, which recovers its fluorescence upon binding of boronic acid to glucose, has been widely reported previously [13,14]. 

Similar approaches have been subsequently developed using luminescent nanoparticles as sensing units. These nanoparticles open prospects for the combination of nanostructure-tuning capabilities with exceptional optical properties and facilitate their application into the cellular environment. For instance, selective glucose nanosensors were obtained when boronic acid was attached onto the surface of graphene nanoparticles, with a sensing mechanism based on aggregation-induced photoluminescence enhancement effects [15], or on the quenching of the nanoconjugate response signal upon binding [16], which allowed the quantification of glucose in brain microdialysates. Carbon-dot [17] and semiconductor quantum dot (QD) nanoparticles [18,19,20,21] are other luminescent nanoparticles that have been functionalized with boronic acid via similar coupling procedures. Even though some of these nanoconjugates permitted the quantification of dopamine [18] or explosives [19] by means of different sensing mechanisms, the more challenging application was the intracellular detection of glucose [17,21]. In such intracellular applications, the analytical signal for the real-time monitoring of glucose levels was a shift in the maximum photoluminescence wavelength of the nanoparticle [21], or, as in the majority of these types of probes, it was based on changes in photoluminescence (PL) intensity when glucose interacted with the nanoparticles. However, these PL intensity-based intracellular measurements can produce misleading readouts because they can be affected by variations in the probe concentration in certain compartments of the cell or by fluctuations in the excitation light. The use of time-resolved fluorescence techniques can overcome many of these limitations, since the PL lifetime is independent of the fluorophore concentration and excitation power [22]. Moreover, fluorescence lifetime imaging microscopy (FLIM) avoids the need for near-infrared probes for intracellular applications, as it can employ fluorophores with emission wavelengths within the range of the green cellular autofluorescence.

Among all these luminescent nanoparticles, the use of time-resolved fluorescence spectroscopy and FLIM is especially advantageous in combination with semiconductor QDs. QDs are an excellent alternative to molecular sensors because their unique optical properties are perfectly appropriate for long-term intracellular monitoring [23]. In particular, QDs show long PL decay times, which are significantly longer than the lifetime of the cell’s autofluorescence, which makes the QDs easily discernible from the intracellular background signal. Additionally, the PL lifetime is highly sensitive to changes in the environment, which allows its use as a detection signal for the development of sensors. Despite the excellent advantages of these kinds of systems, only a few QD-based intracellular nanosensors with FLIM have been reported in the last few years [24,25,26,27,28,29]. 

In this work, we have shown that this approach can be extended to the sensing of glucose in live cells. Even though the FLIM technique has been already used for the study of the response of glucose sensors based on microcapsules [30] or fibre-optics [31] with glucose/galactose-binding proteins labelled with an environmentally sensitive organic fluorophore, this is the first time that semiconductor QD nanoparticles have been employed as a fluorophore, in combination with the FLIM methodology, for the intracellular detection of glucose as the final application. Herein, the aminophenyl boronic acid (APBA) group has been attached to the surface of CdSe/ZnS QDs, producing the quenching of the PL of QD nanoparticles. Upon binding the glucose, the PL is recovered, and the enhancement of the average lifetime of the QD allows the quantification of glucose levels.

## 2. Materials and Methods

### 2.1. Materials

Core-shell CdSe/ZnS QDs with maximum emissions of approximately 520 nm and a lipophilic long chain surfactant capping of octadecylamine (ODA) were purchased from Mesolight (Suzhou, China). In general, all chemicals were used as received without further purification. 3-Mercaptopropionic acid (MPA) was purchased from Fluka. 3-Aminophenylboronic acid (APBA), 1-ethyl-3-(3-(dimethylamino) propyl)carbodiimide hydrochloride (EDC), *N*-hydroxysuccinimide (NHS), phosphate buffer salts, Tris buffer, bovine serum albumin (BSA), and all other inorganic salts were of analytical grade and used as obtained from Sigma-Aldrich (Spain). NaOH (Sigma-Aldrich, Spain) and HCl (Sigma-Aldrich, Spain) (spectroscopic grade quality) were used to adjust the pH of the aqueous (Milli-Q water) solutions and buffers. In order to avoid deterioration by light and heat, stocks solutions were kept in the dark at 4 °C in a refrigerator.

For the cell culture, Dulbecco’s modified Eagle’s medium (DMEM), foetal bovine serum (FBS), sodium pyruvate, penicillin, and streptomycin were obtained from Sigma. GlutaMAX^TM^ supplement was acquired from ThermoFisher (Madrid, Spain). Cell viability assays were carried out using a CellTiter Blue™ viability assay (Promega Biotech Ibérica, Madrid, Spain). For microscopy experiments, all solutions were filtered with 0.2 μm filters (Whatman; as supplied by Sigma-Aldrich, Spain) before use.

### 2.2. Instruments

Steady-state PL spectra were collected at 25 °C using 5 × 10 mm cuvettes in a JASCO FP-6500 (Tokyo, Japan) spectrofluorometer equipped with a 450 W xenon lamp for excitation, with a temperature controller cell holder.

PL decay traces of QDs were recorded in the single-photon timing (SPT) mode using a FluoTime 200 fluorometer (PicoQuant, Berlin, Germany). The samples were excited using a 440 nm pulsed laser (LDH-P-C-440 PicoQuant) with a 10 MHz repetition rate, which was controlled by a PDL-800-B driver (PicoQuant). The full width at half the maximum of the laser pulse was ~80 ps. The PL was collected after crossing through a polarizer set at the magic angle and a 2 nm bandwidth monochromator. The collected photon signals were tagged using a TimeHarp 200 board (PicoQuant), with a time increment per channel of 36 ps. The PL decay traces were collected at emission wavelengths of 518, 520, and 522 nm. The histogram of the instrument response function (IRF) was determined using a LUDOX scatterer. Sample and IRF decay traces were recorded in triplicate until they typically reached 2 × 10^4^ counts in the peak channel.

A MicroTime 200 fluorescence lifetime microscopy system (PicoQuant) was used to recorded the PL lifetime images. The PL decay traces from the QD nanoparticles in the confocal volume were reconstructed with this system, which is based on single-photon timing using the time-tagged time-resolved (TTTR) methodology. The excitation source was a 470 nm pulsed laser (LDH-P-C-470, PicoQuant), operated with a ‘Sepia II’ driver (PicoQuant) set at a repetition rate of 10 MHz. The laser power at the microscope entrance was between 0.2 and 4.4 μW. The excitation beam passed through an achromatic quarter-wave plate (AQWP05M-600, Thorlabs, NJ, USA), set at 45° from the polarization plane of the laser, and was directed by a dichroic mirror (510DCXR, Chroma Technology, as supplied by AHF, Tübingen, Germany) to the oil immersion objective (1.4 NA, 100×) of an inverted confocal microscope (IX-71, Olympus, Tokyo, Japan). The PL emission was collected via the same objective and directed into a 75 μm pinhole by using a dichroic mirror after passing through a specific cutoff, i.e., a long pass filter (500LP, Chroma Technology, as supplied by AHF). The PL emitted photons were detected by using an avalanche photodiode (SPCM-AQR-14, Perkin Elmer, Waltham, MA, USA) after crossing an adequate bandpass filter (600/40, Semrock, as supplied by AHF). Individual photon time tagging was performed within a TimeHarp 200 module (PicoQuant), with a time resolution of 29 ps per channel. To image a region, a sample was raster-scanned with an x-y piezo-driven device (Physik Instrumente, Freiburg, Germany). The imaging data were normally acquired with a 512 × 512 pixel resolution and a collection time of 0.60 ms per pixel.

For the characterization of QD-APBA conjugates FTIR spectra were acquired with a JASCO FT/IR-4600 spectrometer (JASCO, Tokyo, Japan), equipped with an ATR module, and transmission electron microscopy (TEM) images were collected with a LIBRA 120 PLUS transmission electron microscope from Carl Zeiss SMT (Singapore, Singapore).

### 2.3. Synthesis of QD–APBA Conjugates

Initially, lipophilic octadecylamine-capped QDs CdSe/ZnS (QD-ODA) were modified using 3-mercaptopropionic acid (MPA) to achieve water solubility. The procedure for the surface–ligand exchange has been previously reported [32]. Briefly, 1 mL of QD-ODA dissolved in toluene was left to react overnight with 2 mL of MPA, protected from light, to achieve the ligand exchange. Then, 2 mL of 1 M NaOH solution was added, and the mixture was shook in order to transfer the nanoparticles into the aqueous phase. The aqueous phase was separated, and the excess of MPA was removed from the water-soluble CdSe/ZnS QD–MPA nanoparticles by precipitation of the particles with acetone and centrifugation (10 min, 13,000 rpm), followed by the re-dissolution of the QD-MPA in 10 mM Tris buffer, pH 7.5.

**Figure 1:**
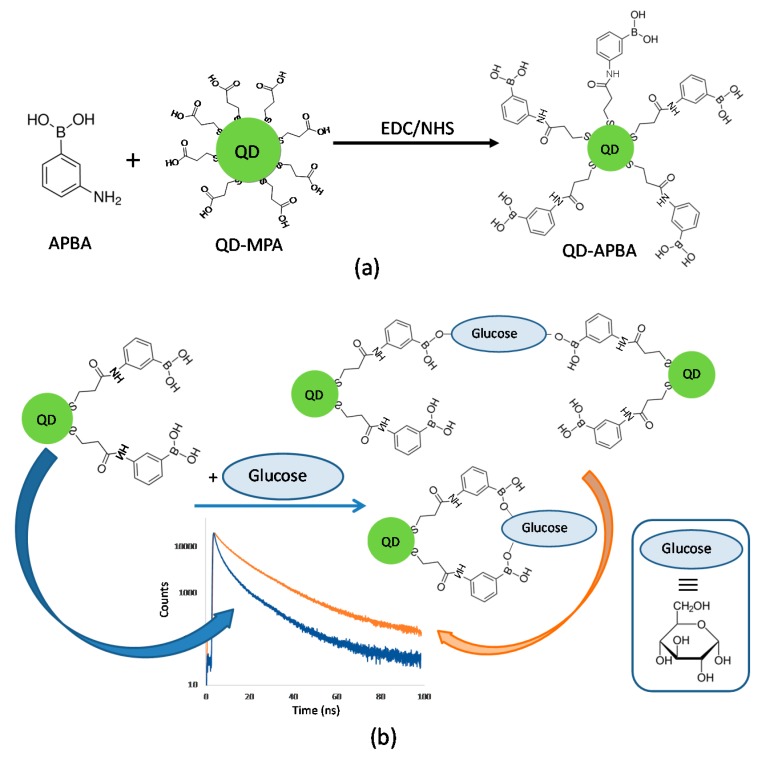
(**a**) Scheme of the covalent coupling reaction for the synthesis of the QD-APBA (quantum dot–aminophenylboronic acid) conjugates. (**b**) Scheme of the proposed mechanism of response of the QD-APBA conjugates toward glucose.

Next, the QD-APBA conjugates were prepared by the reaction of the amino group of the 3-aminophenylboronic acid (APBA) with the carboxylic acid group capping the QD-MPA nanoparticles, thus achieving stable and water-soluble conjugates via amide formation using the EDC/NHS coupling reaction [33] (Figure 1a). Upon optimization of the quantity of 3-Aminophenylboronic acid during the coupling reaction, the QD-APBA conjugates were prepared by mixing 100 μL of QD-MPA with 1 mL of a solution of EDC (100 mM) in 10 mM Tris pH 7.5 for 10 minutes and then with 1 mL of a solution of NHS (50 mM) in 10 mM Tris pH 7.5. After 5 min, 2 mL of a solution of APBA (50 mM) in 10 mM Tris pH 7.5 was added. The mixture was stirred for 3 h at room temperature. The reacting mixture was then centrifuged at 13,000 rpm for 10 min. The supernatant containing the excess of reagents was removed, and the QD–APBA conjugates in the residue were re-dissolved in distilled water.

### 2.4. FLIM Imaging of QD–APBA into MDA-MB-231 Cells 

The breast cancer cell line MDA-MB-231 was obtained from the American Type Culture Collection (ATCC; HTB-26). Cells were grown in DMEM supplemented with 1% GlutaMAX^TM^, 1% sodium pyruvate, 10% (v/v) FBS, and 1% penicillin/streptomycin at 37 °C in a humidified 5% CO_2_ incubator. For the FLIM experiments, MDA-MB-231 cells were seeded onto 20 mm diameter glass slides at a density of 1.35–1.1 × 10^6^ cells/cm^2^. The glass slides were washed with the DMEM medium and phosphate-buffered saline (PBS) before adding the cells. The cells seeded onto the glass slides were incubated for 2 h at 37 °C with the addition of 3 μL of the stock solution of QD–APBA conjugates (containing 75 µg) into 3 mL of the cell culture medium. After incubation, the cells were washed twice with a phosphate buffer at pH 9. For the FLIM experiments, 1 mL of a solution of glucose 50 mM in a phosphate buffer (10 mM) at pH 9 was added to the extracellular medium of the QD-loaded cells and left for the incorporation of glucose into the intracellular medium. Images of the surface areas between 390 and 2450 μm^2^ were collected at different incubation times with a spatial resolution of 10 to 70 nm/pixel.

### 2.5. Cell Viability Assays

The cell viability after incubation with QD nanoparticles was studied by using the CellTiter Blue™ viability assay. To assay the possible side cytotoxicity, cell six repetitions were plated in cell culture-treated, black, 96-well optical flat-bottom plates at 1.2 × 10^3^ cells/well. After 48 h of the cell culture, different amounts of QDs from a sonication-cleared stock solution (1, 2, 4, and 6 µL were added directly to the wells, obtaining 12.5, 25, 50, and 75 µg/mL as final concentrations. For comparison, 25 µg/mL was the concentration used in the FLIM experiments. After 2 h of incubation, 20% v/v of CellTiter-Blue™ reagent was added to the wells and further incubated for 2 h at 37 °C. Then, fluorescence was directly read at 525/580–640 nm in a Glomax®-Multidetection System (Promega Biotech Ibérica, Madrid, Spain). Untreated cell controls and wells with reagents only as background controls were run together with treated cells. The emitted fluorescence was recorded, and, subsequently, the data were expressed as a percentage relative to the untreated control cells.

### 2.6. Methods of Analysis 

The time-resolved PL decay traces collected from experiments in solution were deconvoluted from the instrument response function and fitted using the FluoFit 4.4 package (PicoQuant). The experimental decay traces were fitted to three or tetra-exponential functions via a Levenberg–Marquard algorithm-based nonlinear least-squares error minimization deconvolution method. The quality of the fits was judged by the value of the reduced chi-squared, χ^2^, and visual inspection for random distribution of the weighted residuals and the autocorrelation functions. For the calculation of the intensity-weighted average PL lifetime of QD-MPA and the different QD-APBA conjugates the Equation (1) [34] was used:

(1) $$\tau_{ave}=\sum a_{i}\tau_{i}^{2}/\sum a_{i}\tau_{i}$$

where *τ_i_* denotes the decay times and *a_i_* the corresponding pre-exponential factors. 

FLIM images were analysed using the SymphoTime 32 software (PicoQuant). The FLIM images were reconstructed by sorting all photons corresponding to a single pixel into a temporal histogram via the TTTR methodology. The PL decay traces in the pixels containing the QD emission with a minimum of 200 photons per pixel were fitted to a two-exponential function through an iterative reconvolution method, using the the maximum likelihood estimator (MLE). This methodology obtains the best parameter fitting for low count rates [35]. The short decay time was fixed at 1.5 ns, accounting for the short components and for the cell autofluorescence of the experiments with cells. The second decay time was left as an adjustable parameter. The instrument response function for the iterative reconvolution analysis was reconstructed from images with a high total count rate, using the dedicated routine in the SymphoTime software. To achieve a higher count rate in each pixel, thus improving the reliability of the fits, spatial rebinning of 5 × 5 pixels and temporal binning of four channels in the SPT scale (for a final 116 ps/channel temporal resolution) were employed. The image could then be redrawn using an arbitrary colour scale illustrating just the values of the second, large, decay time in each pixel. 

## 3. Results and Discussion 

### 3.1. Preparation of QD–APBA Conjugates

As is well known, boronic acid moieties act as receptor units for the binding of saccharides. Since boronic acid has been widely employed in glucose sensors, it was expected that the QD modified with APBA groups on their surface (QD-APBA conjugates) would act as photoluminescent glucose nanosensors. For this purpose, first, an optimization of the quantity of conjugated APBA on the surface of the QD nanoparticles was performed. For this, 2 mL of the APBA solutions of different concentrations (5, 20, and 50 mM) in 10 mM TRIS buffer pH 7.5 were added to a fixed quantity of the previously prepared QD–MPA suspension (200 µL), with EDC (100 mM), and NHS (50 mM) to carry out the EDC/NHS coupling reaction. The reaction and purification were performed as explained above (see Materials and Methods section).

**Figure 2:**
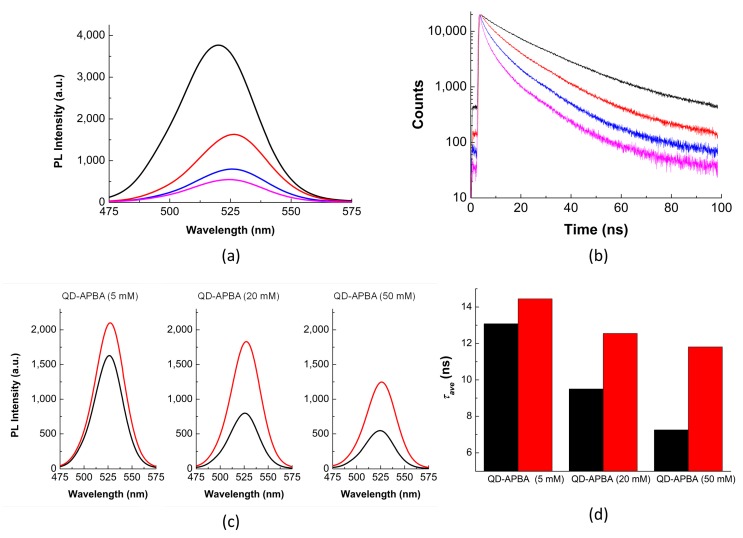
(**a**) Photoluminescence (PL) spectra (λ_ex_ = 440 nm) and (**b**) PL decay traces (λ_ex_ = 440 nm and λ_em_ = 520 nm) of QD–MPA (black) and QD–APBA conjugates synthesized with different amounts of APBA during the coupling reaction: 5 mM (red), 20 mM (blue), and 50 mM (magenta). (**c**) PL emission spectra (λ_ex_ = 440 nm) and (**d**) PL average lifetime of the QD–APBA conjugates synthesized with different amounts of APBA before (black) and after the addition 40 mM glucose (red) (10 mM phosphate buffer, pH 8).

Quenching of the PL intensity of QD-MPA was observed after immobilization of the APBA groups on the surface of the QD nanoparticles. The quenching increased by increasing APBA concentration up to 50 mM. By using a solution of 5 mM APBA during the coupling reaction, the quenching of the PL intensity was ∼60%, whereas 20 mM of APBA produced ∼75% quenching, and 50 mM of APBA caused ∼90% quenching (Figure 2a). Moreover, around a 5 nm red-shift for the maximum emission of the QD nanoparticles was detected after the reaction. The emission maximum of the QD-MPA was 520 nm, but after the formation of each QD-APBA conjugate, the maximum wavelength emission was 525 nm. As it has been reported previously, the binding of ligands at the ZnS shell of the QD nanoparticles may cause a red shift because it may lower the conduction band barrier height and increase the valence band barrier height, thereby resulting in holes to be more localized and electrons to become more delocalized with a small band gap reduction [36]. The shift of the maximum wavelengh observed after the coupling of APBA suggests the successful attachment of the boronic acid group on the surface of the nanoparticles (Figure 2a). The highest concentration of the tested APBA quenched 90% of the PL signal. Therefore, no more APBA was used because these results suggest that no more APBA could be successfully attached onto the surface of the QDs.

PL decay traces of the QD nanoparticles were also collected before and after coupling with the APBA groups. In addition to the changes in the steady-state emission, the PL decay traces of the QD were also modified after assembly (Figure 2b). In agreement with previous reports [28,37,38], the decay traces of the QD-MPA nanoparticles showed tri-exponential behaviour, with long decay times (Table S1 in Supplementary Material). Prior to the immobilization of the APBA group, the intensity-weighted average PL lifetime (*τ_ave_* (see Methods of Analysis)), for the QD-MPA was 19.1 ± 0.1 ns. After the coupling of the APBA group, the decay traces of the QD nanoparticles were fitted to tetra-exponential functions. The four individual decay time components, as well as the average PL lifetime of the QDs, gradually decreased by increasing the concentration of the boronic acid group attached to the surface of the nanoparticles (Table S1). The PL average lifetime of the QD decreased to 13.08 ns, 9.50 ns, and 7.25 ns upon immobilization of the APBA during the coupling reactions with 5, 20, and 50 mM APBA solutions, respectively (Figure 2b, Table S1). This decrease in the average PL lifetime also demonstrates successful coupling of the boronic acid group on the surface of the nanoparticles, which are now available for the binding to glucose molecules. The results of the PL lifetime are in agreement with the patterns observed for the PL intensity of the conjugates (Figure 2).

In addition, the response towards glucose for each batch of QD-APBA with different amount of APBA on the surface was tested. Taking into account that boronic acid can bind monosaccharides such as glucose, we anticipate that the PL properties of the QD-APBA conjugates would change in the presence of glucose when this molecule is bonded to the boronic acid on the surface of the nanoparticles. After the addition of glucose to the QD–APBA conjugates, an increase in the PL emission (Figure 2c) and the PL average lifetime (Table S1, Figure 2d) occurs as a consequence of the disruption of the quenching of the QDs by the APBA group. As an example, Figure 2c,d show the enhancement of the PL intensity and PL average lifetime after the addition of 40 mM glucose to the QD–APBA conjugates prepared with the different amounts of APBA. As can be seen in Figure 2c, by increasing the amount of APBA immobilized on the surface of the QD, the response towards glucose is higher, but this response reaches a plateau when the amount of APBA attached on the surface is higher than 20 mM. However, when the analytical signal is the PL average lifetime, the response is even more prominent for the QD–APBA conjugate synthesized with 50 mM APBA (Table S1, Figure 2d). These results indicate that the sensitivity of the QD–APBA conjugates as glucose nanosensors is directly related to the concentration of the glucose receptor group, the boronic acid attached on the QD. Based on these findings, with the aim of achieving maximum sensitivity, the QD–APBA glucose nanosensors were routinely prepared with 50 mM of APBA. 

**Figure 3:**
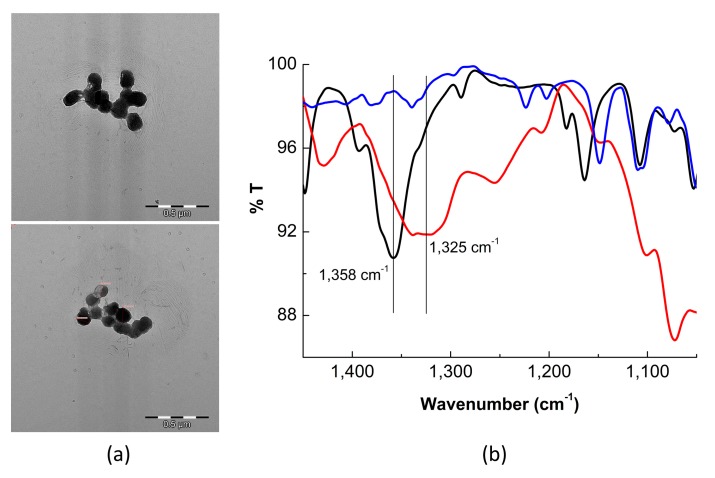
(**a**) TEM images of QD–APBA conjugates. (**b**) FTIR spectra of QD-APBA conjugates in the absence (black) and in the presence (red) of glucose. The FTIR spectrum of glucose (blue) is also added for comparison.

In order to characterize the optimized QD conjugates TEM microscopy and FTIR spectroscopy were used to determine size and morphology of the conjugates, and the success in the attachment of APBA. QD–APBA nanosensors showed spherical shapes, with an average diameter about 100 nm, including the layer of MPA and the consecutive ECD/NHS covalent coupling of the APBA group on the surface of the QD core (Figure 3a). Moreover, the IR spectrum of the QD–APBA conjugates showed the most characteristic bands of phenyl boronic acid. One of the strongest and broadest bands in the spectrum of phenylboronic acid occurs at 1350 cm^−1^, corresponding to the B-O stretching vibrations [39]. Figure 3b shows a band at 1358 cm^−1^, which we assign to this vibration. More interestingly, when glucose is added to the probe this band is shifted to 1325 cm^−1^ as a consequence of the binding of glucose to the B atom. These findings indicate the successful attachment of the boronic group on the surface of the QD nanoparticles and support the proposed response mechanism. 

Next, a study was carried out on the effect of the pH of the medium on the PL features of the QD-APBA and on the glucose response. In these investigations, the PL spectra and decay traces of QD-APBA conjugates in the buffered solutions at different pH values were collected. As previously described, the PL intensity of the QD with carboxylic acids immobilized on the surface are sensitive to pH, since the protonation/deprotonation degree of the carboxylic acids capping the surface of the nanoparticles may alter the confinement of the charge carrier in the semiconductor, with a concomitant change in the PL emission intensity [40] and lifetime [37]. In agreement with this, the emission intensity together with the average lifetime of the QD-APBA conjugates increased gradually with the magnitude of the pH value (Figure S1), as a consequence of the change in the grade of protonation of the free carboxylic acids groups of the MPA remaining on the surface. In addition, the formation of the boronate ion at alkaline conditions [11] may also contribute to the pH sensitivity of the PL intensity of the QD–APBA. 

Nevertheless, it is more interesting to evaluate the pH dependency of the boronic acid–glucose interaction, which is related to the sensitivity of the proposed glucose nanosensor. As is known, boronic acid–diol interactions are highly pH-dependent. Higher pH favours saccharide binding when most of the boronic acid has been transformed to its boronate anion form [11]. Figure S1b shows the PL average lifetime of the QD–APBA conjugates after the addition of 40 mM of glucose in solutions with 10 mM phosphate buffer at different pH values. As expected, the enhancement in the average PL lifetime increased with the pH of the medium, as a consequence of the higher binding of glucose at a more alkaline pH. Thus, in order to obtain the maximum sensitivity in further experiments, the pH of the solutions was fixed to 9 with 10 mM of phosphate buffer. Since the final application of the proposed nanosensor is the intracellular detection of glucose, higher pH values were not considered.

### 3.2. Glucose Response of QD-APBA Nanosensors

**Figure 4:**
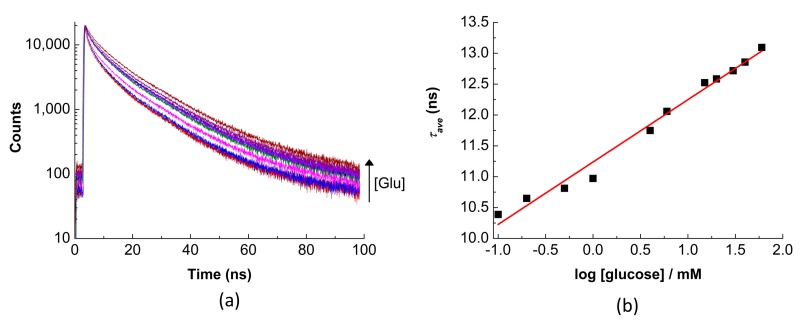
(**a**) PL decay traces (λ_ex_ = 440 nm, λ_em_ = 520 nm) of QD–APBA nanosensors in solutions containing a different concentration of glucose (phosphate buffer 10 mM pH 9). (**b**) Calibration plot: PL average lifetime values calculated from the decays versus the logarithm of the concentration of glucose.

As shown above, the proposed QD-APBA conjugates can act as glucose nanosensors, since the boronic acid groups on the surface of the nanoparticles have the ability to bind monosaccharides. Figure 1b shows a scheme of the proposed response mechanism of QD-APBA conjugates towards glucose. Upon immobilization of the aminophenyl-boronic acid on the surface of the QD nanoparticles, a quenching of the PL was observed (Figure 2). However, when glucose is in the medium, this monosaccharide can be bonded to two boronic acid groups, and the photoluminescent properties were reestablished. Typically, glucose-selective diboronic acid interacts with α-glucofuranose by binding the pair of hydroxyl groups at the 1,2-position and two or three hydroxyl groups of the saccharide at the 3,5,6-position [11]. Nevertheless, in this system, multivalent saccharide binding by two boronic acid groups can be performed in two different pathways: with two boronic acid groups on the surface of the same QD nanoparticle or with two boronic acid groups attached to different QDs (Figure 1b). The result of these interactions is the enhancement of the emission intensity (Figure S2) and of the average lifetime of the conjugates (Figure 4).

The *τ_ave_* of the QD-APBA conjugates showed a dependency on the concentration of glucose with a linear response versus the logarithm of the concentration in a range covering approximately three orders of magnitude, between 0.5 mM and 50 mM of glucose, which suggests their potential application to glucose detection in physiological samples and intracellular media [17,21]. 

The detection limit, estimated as the concentration of glucose that produced an analytical signal equal to three times the standard deviation of the background PL average lifetime [41], was found to be 0.1 mM. This detection limit is similar to other glucose nanosensors based on semiconductor nanoparticles. For instance, previous functionalized core/shell QDs enabled the analysis of glucose by measuring PL intensity with detection limits of 0.1 mM [18], 0.3 mM [21], or 1 mM [7]. More sensitive probes were achieved with graphene quantum dots or with graphene electrodes modified with enzymes. Two different boronic acid functionalized graphene quantum dots nanoparticles were reported, which showed shorter linear response range, about 0.1–10 mM, and detection limits as low as 10 μM [15] or 5 μM [16]. The detection limit was even reduced to 0.5 μM in an electrochemical system in which graphene electrodes were modified with GOx enzyme [10]. Nevertheless, our proposed QD-APBA nanosensors are sensitive enough to glucose concentrations in the typical physiologically important range of 0.4–20 mM [21].

In comparison with other QD-based nanosensors, the range of PL lifetime values (~10–13 ns) and the sensitivity in the variation of the analytical signal of the proposed nanosensor are similar [27,28,29,37]. However, this system is more sensitive than previously published fluorescence lifetime probes based on organic fluorophores [42,43] or fluorescent proteins [44]. Moreover, this sensitivity is also higher than that obtained with other glucose sensors that employ the fluorescence lifetime or FLIM as detection techniques [30,31,45].

Moreover, the temporal behaviour of the PL of the QD nanoparticles was examined by the analysis of the individual lifetime components. As an example, Table S2 gathers the values of the lifetimes and normalized preexponential factors for the QD-APBA conjugates acquired after tetra-exponential fitting of the decay curves found in Figure 4b for measurements in the absence and in the presence of different glucose concentrations. Here, only the slower lifetime components changed after addition of glucose. The two individual longest decay time components were gradually augmented with the medium’s concentration of glucose, while the shorter components were practically unaffected (Table S2). As has been previously reported, the slow decay components are associated with defects on the surface of the nanoparticles [38,46], while the fastest decay components can be attributed to the core-state recombination [47]. The pattern observed in the lifetime components of the QD–APBA conjugates is consistent with processes happening on the surface of the QD nanosensors, and supports the proposed response mechanism in which the binding of glucose to the boronic acid groups on the surface affects the interaction between the QD and boronic acid, with concomitant recovery of the PL emission.

### 3.3. Study of Selectivity

**Figure 5:**
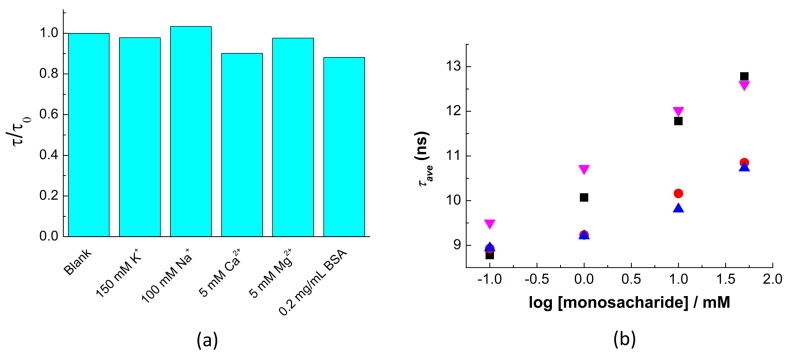
(**a**) Interference study of the QD–APBA conjugates as glucose nanosensors at pH 9. The average PL lifetimes of the QD–APBA nanosensor in the presence of foreign species were normalized by the average PL lifetime of the blank (in the absence of interfering species). (**b**) Average PL lifetime of the QD–APBA nanosensors in the presence of different concentrations of monosaccharides: fructose (black); galactose (red), mannose (blue); glucose (magenta).

The selectivity of the QD-APBA nanosensors was also considered in the presence of the most abundant ions that can be found in cellular cytoplasm and in bovine serum albumin (BSA), which was used to show the potential interactions of proteins with the QD surface and the formation of a protein corona [48]. To study the effect of foreign species, the PL decays of QD-APBA conjugates were collected before and after adding different amounts of these species and were compared. The PL lifetimes of the QD-APBA nanosensor in the presence of foreign species were normalized by the average PL lifetime of the blank (in the absence of interfering species) (Figure 5a). A negligible effect on the QD-APBA PL average lifetime was found when the potential interfering species were in the medium. The absence of important interference shows that these QD-APBA conjugates could be promising candidates as glucose nanosensors in intracellular applications.

Moreover, the response of the QD-APBA nanosensors towards other monosaccharides was also evaluated. Since many saccharides show no structural difference except the configuration of certain stereocentres, boronic acids can bind different saccharides. This makes very hard to discriminate from one another [11]. Generally, the highest binding affinity of phenylboronic acids with monosaccharides is for fructose, followed by galactose, mannose, and glucose, in that order [49], although the selectivity towards glucose can be substantially improved by using multiboronic acid scaffolding. In agreement with other systems based on boronic acid recognition [33,50], the proposed nanosensor showed a response to all the monosaccharides tested. Figure 5b shows that the PL lifetime of the QD-APBA conjugates increases in the presence of glucose, fructose, galactose, and mannose. The maximum sensitivity was obtained for fructose and glucose, with a noticeably lower response for galactose and mannose (Figure 5b). Nevertheless, the low concentrations of fructose, galactose, or mannose that are found in the biological fluids or the cellular cytoplasm lie below the detection limits of our nanosensor, so at these concentrations, these other saccharides should not interfere with glucose determination.

### 3.4. Detection of Intracellular Levels of Glucose

To probe intracellular levels of glucose QD–APBA nanosensors were introduced into live MDA-MB-231 cells. The cells were seeded onto glass slides and then solutions of QD-APBA were added to the culture medium for 2 h at 37 °C. After the incubation time, the MDA-MB-231 cells internalized the nanoparticles, mainly by cellular endocytosis, and maintained their native morphology. Moreover, Figure S2 shows that the QD nanosensors exhibited negligible toxicity on the cells, at least at the concentration ranges and the time frame of the experiments. Once the cytotoxicity of the QD was evaluated the cells were scanned to obtain confocal FLIM images. 

**Figure 6:**
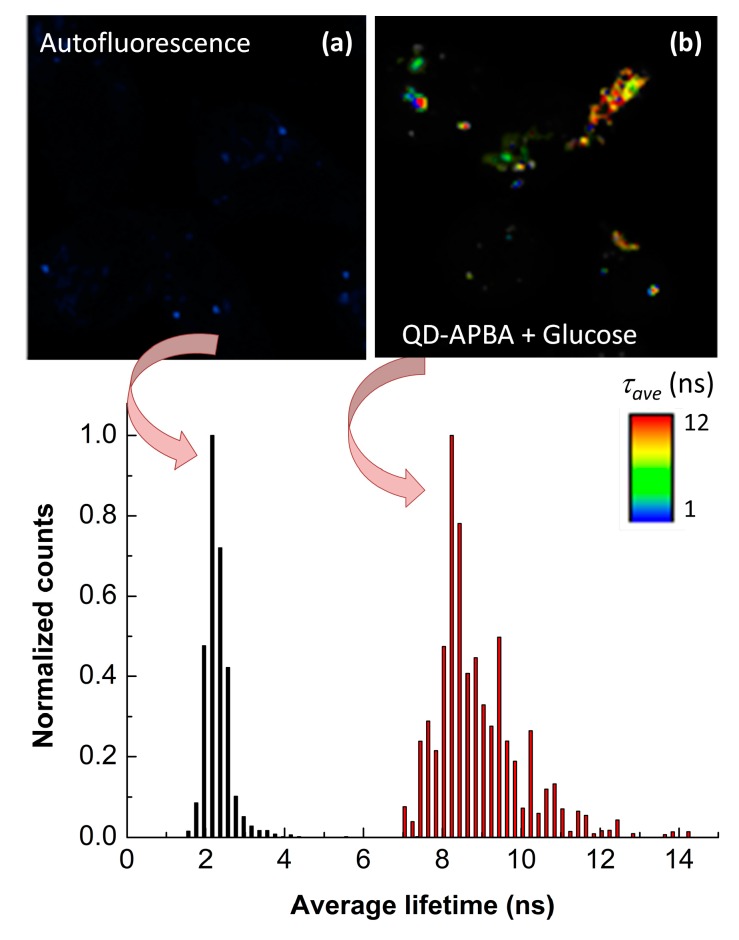
Fluorescence lifetime imaging microscopy (FLIM) images and the corresponding PL lifetime distributions of (**a**) MDA-MB-231 cell autofluorescence and (**b**) MDA-MB-231 cells after 3 h incubation with QD–APBA conjugates and 50 mM glucose.

Figure 6 shows the FLIM images of MDA-MB-231 cells before and after incubation with QD-APBA nanoparticles and 50 mM glucose solution. In the absence of QDs, the cells displayed minimal PL emissions and a uniform distribution of autofluorescence lifetimes across the entire cell, with lifetimes ranging from 1.45 to 2.43 ns, which are similar to the fluorescence lifetimes of many organic dyes. However, the FLIM images of these cells after the uptake of QD-APBA conjugates showed a pronounced contrast between the lifetimes of the nanoparticles and autofluorescence. As indicated by the arbitrary color scale and the lifetime distributions in Figure 6, the PL average lifetime detected from the QDs was significantly longer than that of the cell autofluorescence. These results indicate the excellent characteristics of FLIM microscopy in particularly in combination with QD nanosensors, since they can be distinguished from other interfering fluorescent signal. Moreover, even though the proposed QD–APBA conjugates show PL emissions in the green range of wavelengths, which would overlap with the characteristic green emission of cellular autofluorescence, the use of the PL lifetime as analytical signal facilitates the discrimination between the signal from the nanosensor and the intrinsic fluorescence of the cell. Thus, Figure 6 highlights an enhanced signal-to-background ratio and demonstrates that the combination of FLIM and QD nanoparticles eliminates the requirement for a near-infrared probe in intracellular fluorescence microscopy studies.

**Figure 7:**
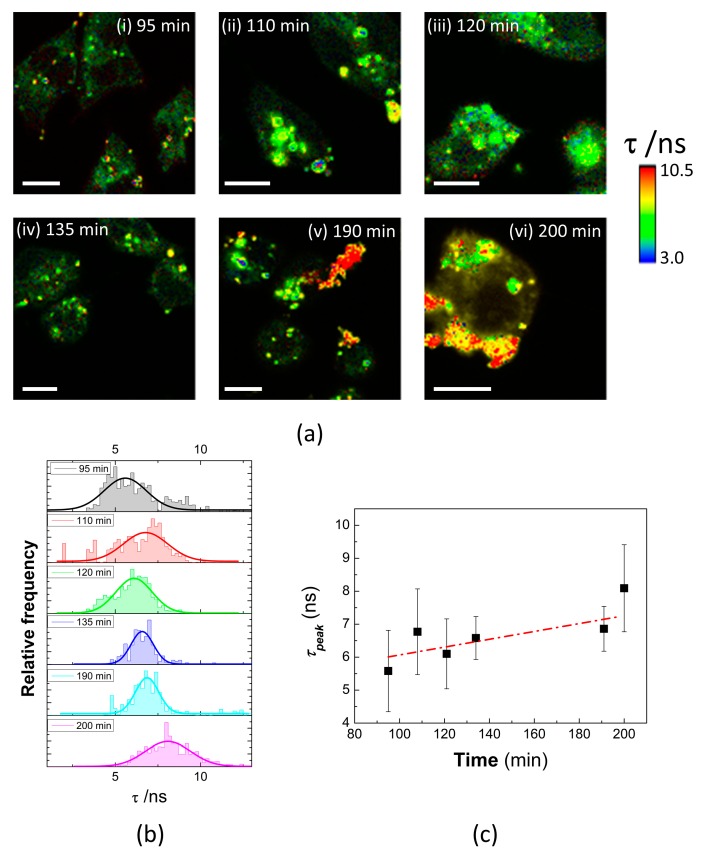
(**a**) FLIM images of QD-APBA conjugates into MDA-MB-231 cells in PBS pH 8.0 before and after addition of a solution of 50 mM glucose to the extracellular medium: (i) 95 min, (ii) 110 min, (iii) 120 min, (iv) 135 min, (v) 190 min, and (vi) 200 min. (**b**) PL Lifetime distributions and (**c**) average PL lifetime calculated from the pixels of interest of the FLIM images in (**a**).

Once the remarkable advantages of the combination of FLIM with QDs for intracellular detection were demonstrated, the usefulness of the QD-APBA conjugates for the intracellular sensing of glucose levels was evaluated in the cytoplasm of MDA-MB-231 cells. To further explore the effect of changes in the intracellular glucose concentration on the PL lifetime of QD-APBA in these cells, the cells were incubated with a solution of 50 mM of glucose (pH 9) and scanned to record the FLIM images at different times. Figure 7 shows the changes in the PL lifetime of QD-APBA in the cytoplasm of MDA-MB-231 cells treated with glucose at different times of incubation. Direct visualization of the changes in the PL lifetime of the QDs can be seen in the arbitrary color scale of the FLIM images in Figure 7a. The PL average lifetime calculated from the analysis of the regions of interest (pixels with QD emission detected) increased with the incubation time, as a consequence of the internalization of glucose from the extracellular media and the concomitant augmentation in the intracellular glucose concentration (Figure 7b). These results suggest the excellent response of the proposed QD-APBA lifetime-based glucose nanosensors inside the cells and that they are capable of measuring changes in the dynamic intracellular glucose concentration. This approach is particularly interesting for intracellular sensing, because of the advantages of the time-resolved fluorescence techniques described earlier in this paper.

## 4. Conclusions

In conclusion, we have demonstrated that the use of QD-based photoluminescent nanosensors is a good alternative to conventional electrochemical glucose probes based on glucose oxidase or other enzymes, which have limited accuracy and reduced responses in vivo, possibly because they suffer from some interference caused by the presence of electroactive substances in the tissues, among others. 

We have proven that glucose nanosensors can be prepared by the covalent linking of aminophenylboronic acid on the surface of CdSe/ZnS core/shell QD nanoparticles. The working mechanism of our QD-APBA conjugates is based on the typical binding capacity of the boronic acid moiety to monosaccharides and is discussed in our work. Since boronic acid can complex glucose with a high binding constant, the proposed QD–APBA conjugates offer a good system to investigate glucose levels. The quenching observed in the PL average lifetime of QD by NHS/EDC coupled phenylboronic acid on the surface can be reversed by exposure of the conjugates to glucose, stopping the interaction with the QD, thus “turning on” the emission of the QD nanoparticles, with the concomitant enhancement of the PL lifetime. 

Importantly, the system showed good sensitivity towards glucose in the range of physiological levels, and the suitability of these glucose-sensitive QDs for the detection of changes in the intracellular levels of glucose into MDA-MB-231 cells was also demonstrated with FLIM. Previous reports have shown different modified QDs and other fluorescent nanoparticles applied as glucose nanosensors, although the detection signal has been always the luminescence intensity, which presents several weaknesses, especially for intracellular quantitative measurements. Here, we have shown the benefits of the FLIM methodology, principally in combination with QD nanoparticles, whose very long PL lifetime significantly enhances the sensitivity and selectivity of the nanosensors. Moreover, these long decay times enable the discernment between the signal from the sensor and the intrinsic cellular autofluorescence, thereby producing a greater signal-to-background ratio.

Nonetheless, these excellent advantages have not yet been exploited, since, in the last few years, only a few examples of studies using FLIM microscopy with QD-based nanosensors have been reported [25,27,28,29]. In this work, we have extended this methodology to the development of a robust nanosensor for the detection of a molecule of high biological interest. Changes at the glucose level within cells can be indicative of many cellular processes and play an important role in both energy metabolism and biosynthesis in the cells. Thus, taking into account the importance of the intracellular levels of glucose and its relationship to the metabolic status of cancer cells, the proposed nanosensors can potentially be used in cancer diagnosis.

## Supplementary Materials

The following are available online at https://www.mdpi.com/1424-8220/19/22/4992/s1: Figure S1: Effect of pH; Figure S2: Changes in the PL intensity; Tables S1–S2: Decay times and normalized pre-exponentials of QD nanoparticles; Figure S3: Cytotoxicity.
