# Supplementary material for: A Quantum Dot-Based FLIM Glucose Nanosensor
Source: Sensors (Basel). 2019 Nov 16;19(22):4992. doi: 10.3390/s19224992 (PMC6891378; doi:10.3390/s19224992)
Supplement: Supplementary file 1 [file sensors-19-04992-s001.pdf]

sensors

Supplementary Materials

Article

A Quantum Dot-Based FLIM Glucose nanosensor

Consuelo Ripoll, Angel Orte, Lorena Paniza and Maria Jose Ruedas-Rama*

Dept. Physical Chemistry. Faculty of Pharmacy. Unidad de Excelencia de Química Aplicada a la Biomedicinay Medioambiente (UEQABM). University of Granada. Campus Cartuja, 18071 Granada (Spain)
* Correspondence: mjruedas@ugr.es; Tel.: +34-958-247887

Effect of pH
- **Figure S1………………………………………………... S1**

Changes in the PL intensity
- **Figure S2………………………………………………... S2**

Decay times and normalized pre-exponentials of QD nanoparticles:
- **Table S1………………………………………………… S2**
- **Table S2………………………………………………… S3**

Cytotoxicity:
- **Figure S3……………………………………………... S4**

Effect of pH

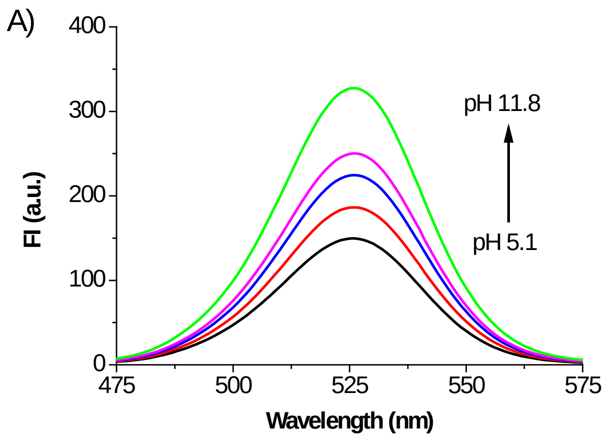

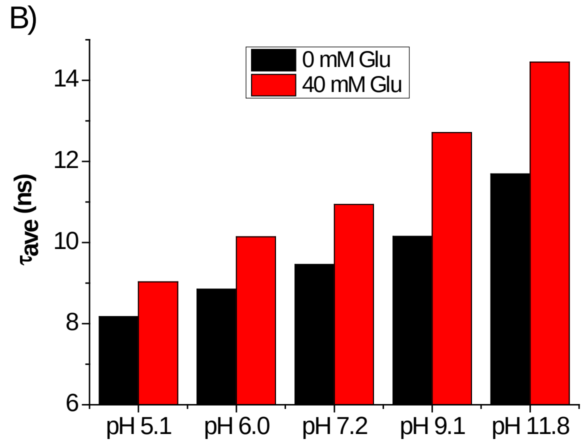

Supplementary Figure S1. A) PL emission spectra of QD-APBA conjugates in 10 mM phosphate buffer solutions at different pH values. B) PL average lifetime of QD-APBA conjugates in 10 mM phosphate buffer solutions at different pH values, in the absence (black) and in the presence (red) of 40 mM Glucose.

Changes in the PL intensity

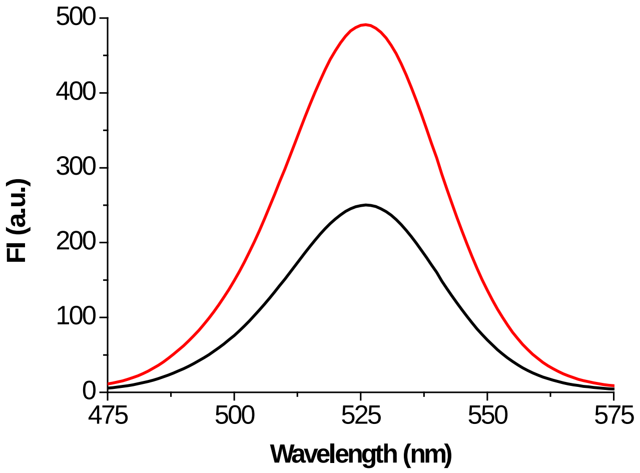

Supplementary Figure 2. PL emission spectra of QD-APBA conjugates in 10 mM phosphate buffer solution at pH 9 before (black) and after addition 40 mM glucose (red).

Decay times and normalized pre-exponentials of QD nanoparticles

To investigate the temporal behaviour of the photoluminescence of the QD nanoparticles, the individual lifetime components were examined.

Supplementary Table 1. Decay times and normalized pre-exponentials of QD-MPA and QD-APBA conjugates prepared with different amounts of APBA during the coupling reaction (re-suspended in 10 mM phosphate buffer pH 9). PL decay traces collected at λ_{ex} = 440 nm and λ_{em} = 520 nm.

	(a_1) τ_1 (ns)	(a_2) τ_2 (ns)	(a_3) τ_3 (ns)	(a_4) τ_4 (ns)	τ_{ave} (ns)[a]	χ^2
**QD-MPA**	(0.46) 22.82	(0.41) 11.58	(0.13) 1.77	-	19.07	1.286
**QD-APBA (5 mM APBA)**	(0.14) 20.78	(0.46) 9.79	(0.25) 2.76	(0.16) 0.64	13.08	1.193
**QD-APBA (20 mM APBA)**	(0.08) 17.61	(0.30) 7.44	(0.32) 2.20	(0.30) 0.51	9.50	1.145
**QD-APBA (50 mM APBA)**	(0.03) 16.92	(0.16) 6.68	(0.32) 1.97	(0.48) 0.50	7.25	1.109
**QD-APBA (5 mM APBA) + 40 mM Glu**	(0.24) 20.32	(0.47) 10.11	(0.18) 2.98	(0.11) 0.89	14.45	1.241
**QD-APBA (20 mM APBA) + 40 mM Glu**	(0.18) 18.82	(0.40) 8.93	(0.26) 2.56	(0.16) 0.61	12.55	1.201
**QD-APBA (50 mM APBA) + 40 mM Glu**	(0.16) 18.00	(0.33) 8.20	(0.28) 2.42	(0.23) 0.63	11.81	1.173

[a] Associated errors in τ_{ave}, obtained through error propagation of the fitting errors of the adjustable parameters, were always between 0.13 and 0.18 ns.

Supplementary Table 2. Decay times and normalized pre-exponentials of the optimized QD-APBA nanosensor at different glucose concentrations in phosphate buffer 10 mM pH 9. PL decay traces collected at λ_{ex} = 440 nm and λ_{em} = 520 nm.

	**(a_1) τ_1 (ns)**	**(a_2) τ_2 (ns)**	**(a_3) τ_3 (ns)**	**(a_4) τ_4 (ns)**	**τ_{ave} (ns)[a]**	χ^2
**QD-APBA**	(0.10) 17.06	(0.25) 7.55	(0.28) 2.45	(0.37) 0.66	9.94	1.168
**QD-APBA + 0.1 mM Glucose**	(0.12) 16.39	(0.25) 6.93	(0.27) 2.14	(0.35) 0.59	10.28	1.166
**QD-APBA + 0.2 mM Glucose**	(0.11) 17.40	(0.27) 7.71	(0.28) 2.44	(0.34) 0.66	10.45	1.157
**QD-APBA + 0.5 mM Glucose**	(0.12) 17.06	(0.26) 7.66	(0.26) 2.45	(0.37) 0.67	10.58	1.147
**QD-APBA + 1 mM Glucose**	(0.12) 17.44	(0.27) 7.59	(0.36) 2.38	(0.25) 0.69	10.86	1.200
**QD-APBA + 4 mM Glucose**	(0.14) 17.91	(0.29) 8.00	(0.27) 2.33	(0.30) 0.62	11.47	1.191
**QD-APBA + 6 mM Glucose**	(0.16) 18.04	(0.31) 8.06	(0.27) 2.35	(0.26) 0.64	11.88	1.212
**QD-APBA + 15 mM Glucose**	(0.23) 18.20	(0.09) 8.33	(0.33) 2.51	(0.35) 0.65	12.28	1.220
**QD-APBA + 20 mM Glucose**	(0.17) 18.44	(0.32) 8.74	(0.26) 2.67	(0.25) 0.65	12.42	1.196
**QD-APBA + 30 mM Glucose**	(0.19) 18.16	(0.34) 8.13	(0.25) 2.30	(0.23) 0.60	12.54	1.204
**QD-APBA + 40 mM Glucose**	(0.18) 18.73	(0.35) 8.60	(0.25) 2.55	(0.23) 0.67	12.70	1.257
**QD-APBA + 60 mM Glucose**	(0.21) 18.63	(0.36) 8.57	(0.24) 2.42	(0.19) 0.63	13.13	1.236

[a] Associated errors in τ_{ave}, obtained through error propagation of the fitting errors of the adjustable parameters, were always between 0.11 and 0.17 ns.

The differences between the PL lifetimes of each component and the concomitant average lifetimes are due to batch to batch variations. The Quantum Dots (CdSe/ZnS core-shell) nanoparticles used in this work were commercially available and were purchased from Mesolight (USA). Originally, the QDs showed maximum emissions of approximately 520 nm and had octadecylamine (ODA) as lipophilic long chain surfactant capping. During the performance of the experiments of this work, different batches of QD-ODA were employed, which showed slightly different photoluminiscent properties. Even though the PL average lifetime of the QD-APBA conjugates were different when the QD-ODA came from different batches, the relative response to glucose, and therefore, the sensibility of the proposed nanosensors, was similar (see Tables S1 and S2). Only two batches were employed: the experiments for the optimization of the QD-APBA conjugates were performed with one batch (Table S1), and the rest of studies (calibration, interferences studies, intracellular applications) were carried out with a second batch.

Cytotoxicity

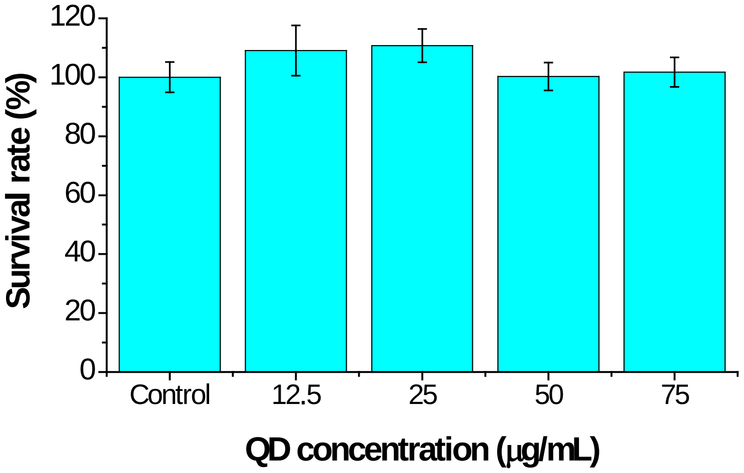

Supplementary Figure 3. Survival rate of MDA-MB-231 cells upon 2-hour incubation with QDs conjugates at different dosages corresponding to ×0.5, ×1, ×2, and ×3 times the concentration of QDs used in the cell FLIM imaging experiments. Error bars are expressed as s.e.m. from 6 repetitions.
